# Enhanced Visible Light Controlled Glucose Photo-Reforming Using a Composite WO_3_/Ag/TiO_2_ Photoanode: Effect of Incorporated Plasmonic Ag Nanoparticles

**DOI:** 10.3390/nano14242001

**Published:** 2024-12-13

**Authors:** Katarzyna Jakubow-Piotrowska, Bartlomiej Witkowski, Piotr Wrobel, Krzysztof Miecznikowski, Jan Augustynski

**Affiliations:** 1 Centre of New Technologies, University of Warsaw, S. Banacha 2c, 02-097 Warsaw, Poland; 2 Faculty of Chemistry, University of Warsaw, Pasteura 1, 02-093 Warsaw, Poland; bwitk@chem.uw.edu.pl (B.W.); kmiecz@chem.uw.edu.pl (K.M.); 3 Faculty of Physics, University of Warsaw, Pasteura 5, 02-093 Warsaw, Poland; pwrobel@igf.fuw.edu.pl

**Keywords:** photoelectrochemical oxidation, glucose conversion, silver catalyst, visible light absorption

## Abstract

WO_3_/Ag/TiO_2_ composite photoelectrodes were formed via the high-temperature calcination of a WO_3_ film, followed by the sputtering of a very thin silver film and deposition of an overlayer of commercial TiO_2_ nanoparticles. These synthetic photoanodes were characterized in view of the oxidation of a model organic compound glucose combined with the generation of hydrogen at a platinum cathode. During prolonged photoelectrolysis under simulated solar light, these photoanodes demonstrated high and stable photocurrents of ca. 4 mA cm^−2^ due, on one hand, to the occurrence of the so-called photocurrent doubling and, on the other hand, to the plasmonic effect of Ag nanoparticles. The post-photoelectrolysis analyses of the electrolyte demonstrated the formation of high-value final glucose photo-reforming products, principally gluconic acid, erythrose and formic acid.

## 1. Introduction

Water splitting is regarded as a sustainable way of producing hydrogen by absorbing and transferring the energy from photons in sunlight. Shortly after the beginning of investigations into the production of hydrogen via photoelectrochemical water splitting, first reported by Fujishima and Honda [1], it was noted that the overall efficiency of the process is controlled by the sluggish counterpart oxygen evolution reaction occurring at the TiO_2_ photoanode. This observation prompted a series of studies on other more kinetically favorable substrates able to replace water oxidation. Apparently, the first such work was by Kawai and Sakata, who reported [2] using a RuO_2_/TiO_2_/Pt photocatalyst, carrying out the oxidation of saccharose and starch into CO_2_ with simultaneous hydrogen evolution. In such a system, the RuO_2_/TiO_2_/Pt particles operated as photoelectrochemical micro-cells with RuO_2_/TiO_2_ playing the role of a photoanode and Pt/TiO_2_ that of a cathode. That work demonstrated, for the first time, the preferential photo-oxidation of polysaccharides and, consequently, of glucose, promoting hydrogen generation both in photocatalytic and photoelectrochemical systems.

Practically, glucose can be obtained from agricultural waste biomass through hydrolysis of its major constituent cellulose and hemicellulose [3,4,5]. The possibility of conducting the conversion of glucose into fine organic products under milder reaction conditions than those required by selective catalytic glucose oxidation prompted a large number of investigations on its photo-reforming. The major part of photo-reforming studies relies on the use of photocatalysts in the form of suspensions of n-type semiconductor nanoparticles [2] where photo-oxidation and reduction reactions take place on the same particle. In most cases, these studies employed TiO_2_ and modified TiO_2_ photocatalysts able to capture UV and, in general, only a marginal portion of visible light [6,7,8,9,10,11,12,13,14]. Similarly, modified TiO_2_ results were reported using Pt-decorated NiTiO_3_ [15]. An important part of the work regarding photocatalytic processes applied to the conversion of biomass (including glucose) into value-added organic products has been recently reviewed by de Assis et al. [16].

Quite generally, a major problem associated with the use of photocatalyst suspensions with large surface areas is a slow reaction rate and the difficulty of separating the adsorbed final reaction products from the photocatalyst. In order to facilitate product desorption from the photocatalyst surface and increase selectivity, a mixture of water and polar aprotic solvent (acetonitrile) is used in some cases [9,17], which generates environmental problems. Finally, a mixture of photo-oxidation and reduction products is obtained in the unique compartment of the photocatalytic cell. The situation is different in a photoelectrochemical (PEC) system where the photoanode and the (photo)cathode are placed in two different cell compartments separated by a membrane. Consequently, the reduction products, such as hydrogen, may be collected independently in the cathode compartment. In contrast with the photocatalyst suspensions, the PEC configuration also offers the possibility of controlling the photoanodic process separately, e.g., by modifying the external bias voltage imposed on the PEC cell [18].

Only a limited number of published works have been devoted to glucose photo-reforming in PEC systems [19]. Esposito et al. [20], using thin-film WO_3_ photoanodes, observed a relatively low extent of glucose conversion into CO_2_, i.e., its total mineralization. However, intermediate oxidation products could not be identified by the authors. In a very recent work, Tian et al. [21], who employed black hydrogenated TiO_2_ [22] nanorods decorated with “single atom” platinum formed by atomic layer deposition, reported efficient glucose photo-reforming into mainly glucaric acid under simulated sunlight irradiation. Apparently, the performance of the Pt/def.TiO_2_ photoanode could be maintained over five repeated 5.5 h long glucose photoelectrolysis tests. However, despite the electrochemical reduction treatment of TiO_2_ nanorods leading to their black coloration, the effective absorption of visible light by the obtained Pt/def-TiO_2_ photoanodes appeared limited to ca. 430 nm, as shown by the reported incident photon-to-glucaric acid conversion efficiency (IPCE) spectrum.

In the present work, we investigated the oxidation of glucose into value-added organic products using a composite photoanode, consisting of mesoporous WO_3_ covered with a very thin Ag film and with an overlayer of TiO_2_ nanoparticles, operating in a PEC cell. We explored, in particular, the plasmonic effect of Ag nanoparticles, as well as the role of the composition of the employed supporting electrolyte. The fabricated composite photoanodes exhibit enhanced visible light absorption, rapid electron/hole separation, and hole transfer towards the electrode–solution interface.

## 2. Materials and Methods

### 2.1. Synthesis of WO_3_ Mesoporous Film

**Figure 1:**
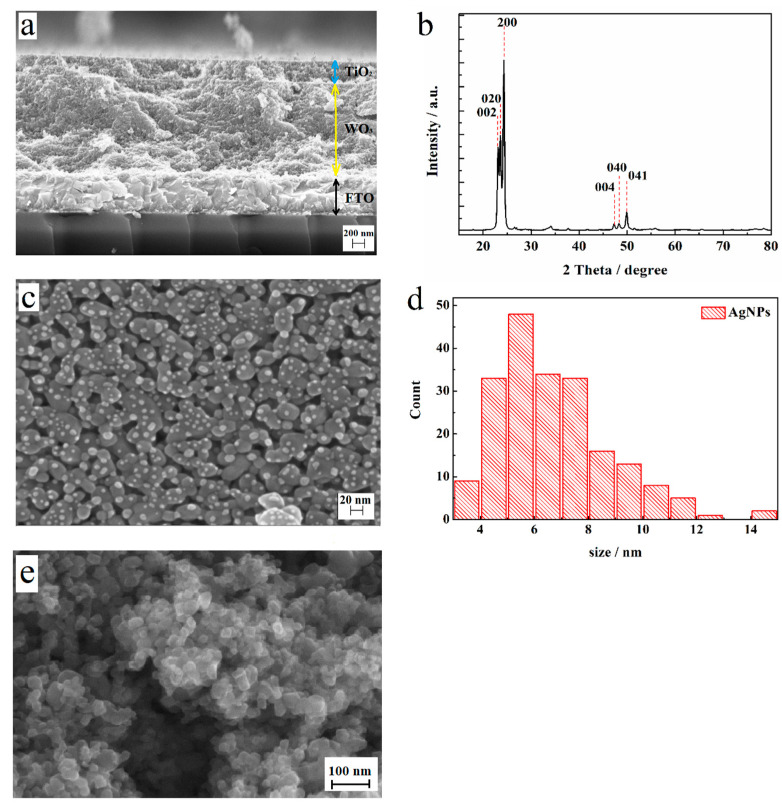
(**a**) A cross-sectional SEM image of the WO_3_/Ag/TiO_2_ film electrode. (**b**) The X-ray diffraction pattern of a WO_3_ film annealed at 550 °C with a perfect monoclinic crystalline structure. (**c**) A top-view SEM image of the WO_3_ film covered with silver nanoparticles. (**d**) A particle size distribution histogram of Ag NPs. (**e**) A top-view SEM image of the WO_3_/Ag/TiO film.

**Scheme 1:**
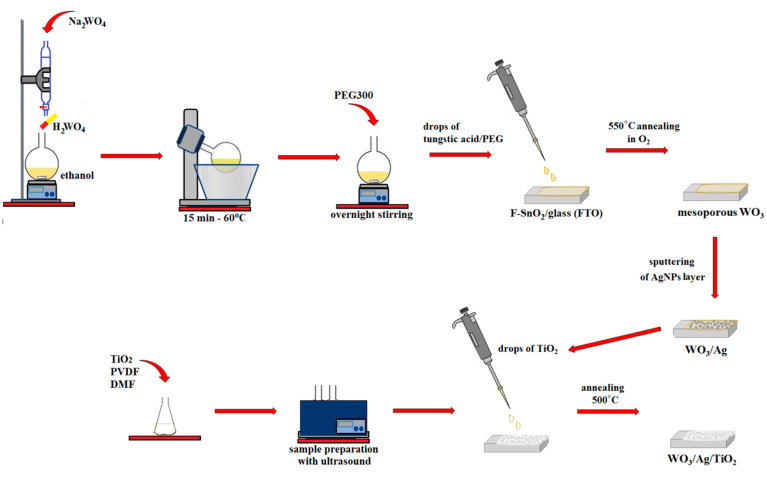
The preparation process of WO_3_/Ag/TiO_2_ film electrodes used in glucose photo-reforming experiments.

The WO_3_ films were prepared based on fresh tungstic acid using a sol–gel method. In brief, 0.5 M aqueous solution of Na_2_WO_4_ was eluted through a proton exchange resin: Dowex 50 WX2-200 (Saint Louis, MO, USA). The solution was collected in ethanol to prevent the condensation of tungstic acid to form polyoxoanions. After elution was complete, the solution was concentrated under vacuum to around ∼0.5 M before the addition of polyethylene glycol (PEG) 300 and kept under continuous stirring. The WO_3_/PEG ratio used in the precursor was 0.4 *w*/*w*. The films were obtained by a sequential layer-by-layer deposition and annealing method. The creation of a complex between H_2_WO_4_ and the hydrophilic polyethylene glycol delayed the formation of fully crystallized monoclinic tungsten trioxide below a temperature of ∼500 °C. The precursor solution was coated via the doctor blading method and briefly air-dried onto the F-doped SnO_2_ (FTO)-covered glass substrates, provided by Sigma Aldrich, at a resistance of 7 ohms/square. Then, films were annealed in the flow of oxygen for 30 min at 550 °C. The process of WO_3_ film preparation is presented in Scheme 1. The WO_3_ films used in this work had three layers of precursor solution, and each of them was annealed in O_2_ at 550 °C for 30 min [23]. The WO_3_ film final thickness employed to build hybrid photoanodes was ca. 1.4 µm, as determined by cross-sectional scanning electron microscopy (SEM), as shown in Figure 1a.

### 2.2. Preparation of Silver Nanoparticles

Silver nanoparticles were fabricated via the deposition of thin films by the electron beam physical vapor deposition technique (e-PVD, Lesker PVD 75, Pittsburgh, PA, USA). A thickness of 1 nm was chosen to form well-separated semi-spherical particles. The thickness and the evaporation rate of the layers were measured during the evaporation process with the use of two quartz crystal microbalance sensors (QCMs, Inficon, 6 MHz gold-coated crystals, Bad Ragaz, Switzerland) placed close to the crucible and the sample, respectively. The sensors were calibrated prior to the experiment. Silver catalyst formed islands with sizes ranging from a few to more than 10 nm, as shown on the SEM image in Figure 1c. The particle size distribution histogram of silver nanoparticles is presented in Figure 1d.

### 2.3. The TiO_2_ Overlayer

Subsequently, the WO_3_/Ag film was covered with a ca. 0.3 μm thick layer of P25 TiO_2_ (from Ovonics) nanoparticles, deposited following the previously described method [24]. The TiO_2_ layer of WO_3_/Ag/TiO_2_ photoanode is shown in the cross-sectional SEM image in Figure 1a and in the top-view SEM image in Figure 1e. Both images show a three-dimensional structure composed of TiO_2_ crystals of different sizes.

All SEM images, as well as the morphology of the silver-coated samples, were investigated using a field emission scanning electron microscope (SEM, Zeiss, Sigma HV, Oberkochen, Germany) equipped with the in-lens secondary electrons detector. The X-ray powder diffraction (XRD) and pattern of the WO_3_ films were obtained by a Siemens D500 diffractometer equipped with a high-resolution semiconductor Si/Li detector using Cu Kα radiation.

The photocurrent-potential (j-E) and incident photon-to-current conversion efficiency (IPCE) measurements were performed in a Teflon cell equipped with a quartz window (with 45 mL of anolyte). The composite photoanode was illuminated through the FTO substrate (exposed surface area 0.28 cm^2^). The simulated AM 1.5G (100 mW cm^−2^) illumination was obtained from an Oriel 150 W solar simulator. All electrochemical experiments were measured in a three-electrode system with a composite photoanode, separated by a Nafion membrane from a platinum grid cathode, and a reference electrode (Ag/AgCl, E = 0.197 V vs. SHE). The incident photon-to-current conversion efficiency measurements of the photoanodes were obtained using light from a 150 W xenon lamp passing through a photoelectric spectrometer featuring a monochromator with a bandwidth of 10 nm. The products of glucose photo-reforming were identified after the long-term light exposition (5 to 20 h) of the composite photoanode (ca. 0.9 cm^2^ surface area).

All chromatographic analyses were performed with a GC-MS-QP2010 Ultra gas chromatograph interfaced with a single quadrupole mass spectrometer equipped with AOC-5000 autosampler manufactured by Shimadzu, Canby, OR, USA. GCMS solution v4.41 software was used for data acquisition and processing.

The mineralization of glucose was monitored with a TOC-5050A total organic carbon analyzer connected with an ASI-5000A autosampler manufactured by Shimadzu, Kyoto, Japan.

## 3. Results

The consecutive steps involved in the WO_3_/Ag/TiO_2_ film preparation process are shown in Scheme 1.

Figure 1 presents the morphological (Figure 1a,c,d) and structural (Figure 1b) characteristics of the formed film. On the cross-sectional SEM image (Figure 1a), we can clearly identify the FTO substrate, the WO_3_ film, and the TiO_2_ overlayer. However, due to the use SEM magnification, the silver inter-layer cannot be distinguished. The SEM image in Figure 1c shows the typical morphology of the WO_3_ film composed of partly fused NPs with sizes between 30 and 50 nm and numerous pores of similar sizes. To achieve a quasi-spherical shape, the nano-island films were annealed at 150 °C for 5 min on a standard hot plate. The XRD pattern in Figure 1b for the monoclinic WO_3_ film reveals dominant (002), (020), and (200) peaks, as well as less intense (004), (040), and (041) peaks, indicating the preferential orientation of the crystallites parallel to the FTO substrate. Figure 1c also shows the layer of silver NPs deposited by sputtering on the surface of the WO_3_ film. As indicated by the histogram in Figure 1d, the major part of Ag NPs has sizes in the range of 4–8 nm.

**Figure 2:**
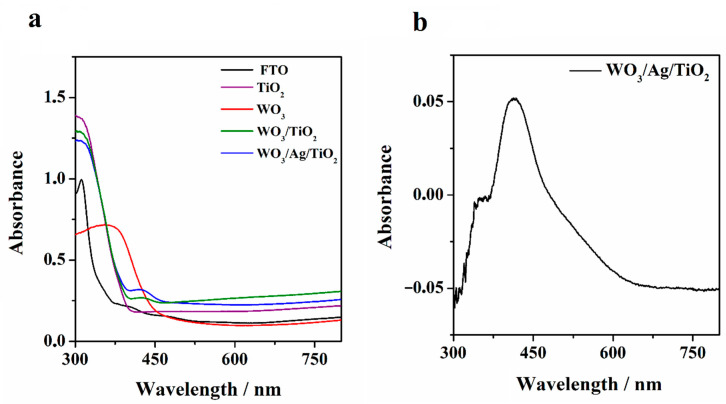
(**a**) Absorbance spectra of FTO substrates: bare (black), with a TiO_2_ layer (violet), with a WO_3_ layer (red), with a TiO_2_ and WO_3_ bilayer (green), and with Ag nanoparticles placed between the WO_3_ and TiO_2_ layers. (**b**) Relative absorbance spectra of the Ag-enhanced photoanode related to the reference FTO/WO_3_/TiO_2_ sample.

Figure 2 shows the absorbance spectra of FTO substrates covered with additional constituent layers depicting their input to the overall absorptivity of the working photoanode. The addition of Ag nanoparticles at the WO_3_/TiO_2_ interface resonantly increases absorbance at around 430 nm, proving the excitation of the localized surface plasmons. The resonant nature of the phenomenon is clearly seen when presented as a relative absorbance related to the reference FTO/WO_3_/TiO_2_ sample that is presented in Figure 2b. The increased slope around 500 nm comes from the asymmetric size distribution of the nanoparticles shown in Figure 1d, while the short-wavelength side of the curve is also affected by the absorption edge of the TiO_2_. It is worth mentioning that the parameters of the resonance, like spectral position, height, and width, depend on the material composition, size, and size distribution of the nanoparticles, which can be controlled simply by the nominal thickness of the deposited layers and the evaporation conditions.

**Figure 3:**
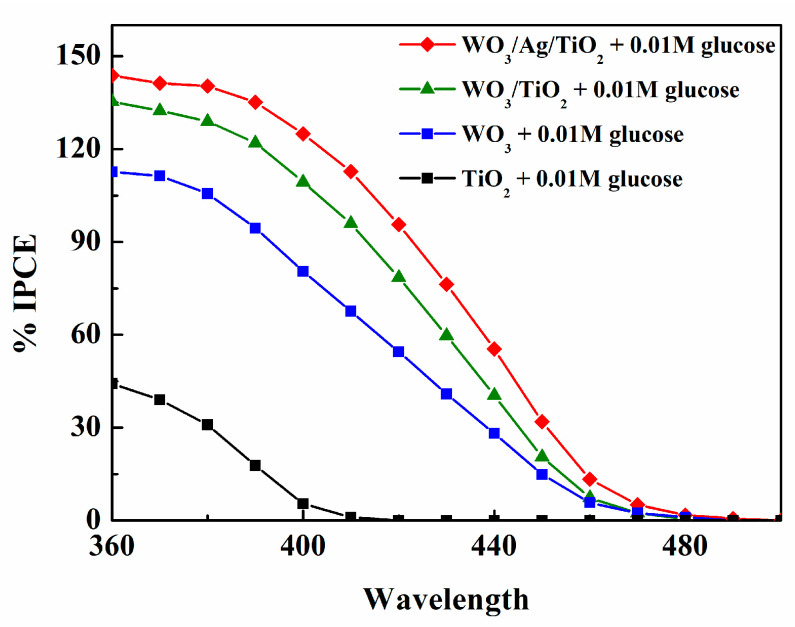
IPCE spectra for WO_3_, TiO_2_, WO_3_/Ag/TiO_2_, and WO_3_/TiO_2_ photoanodes measured at 0.6 V vs. Ag/AgCl in a 0.01 M NaCl/Na_2_SO_4_ electrolyte of pH 7 containing 0.01 M glucose.

In order to highlight the role of silver nano-catalyst, the photoelectrochemical characterization and prolonged glucose photoelectrolysis experiments have been performed for the WO_3_/Ag/TiO_2_ photoanode, as well as for a similar hybrid photoanode, WO_3_/TiO_2_, without silver and for bare WO_3_ and TiO_2_. Figure 3 represents the IPCE spectra measured for four electrodes in the solution of the NaCl/Na_2_SO_4_ supporting electrolyte with 0.01 mol L^−1^ of glucose. We note significantly larger IPCEs for the WO_3_/Ag/TiO_2_ electrode over the whole 350–490 nm range of wavelengths. The fact that both curves, WO_3_/Ag/TiO_2_ and WO_3_/TiO_2_, are almost parallel may suggest that the presence of silver enhances light reflection by the TiO_2_ overlayer and, at the same time, acts as an electrocatalyst, reducing charge carrier recombination at the surface of WO_3_.

**Figure 4:**
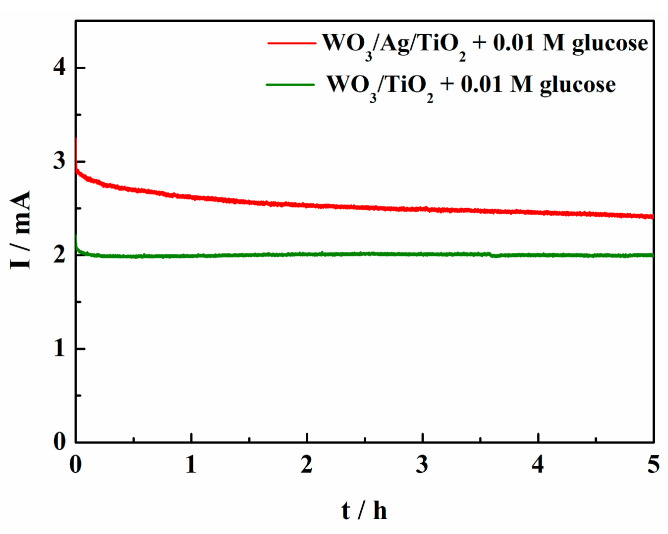
The photo-currents of glucose oxidation measured during 5 h long electrolysis conducted at 0.6 V vs. Ag/AgCl in a 0.01 M NaCl/Na_2_SO_4_ electrolyte of pH 7, using WO_3_/Ag/TiO_2_ and WO_3_/TiO_2_ photoanodes irradiated with simulated AM 1.5 G solar light.

The latter hypothesis is consistent with the photocurrent vs. time plots recorded during 5 h long electrolysis of glucose shown in Figure 4.

**Figure 5:**
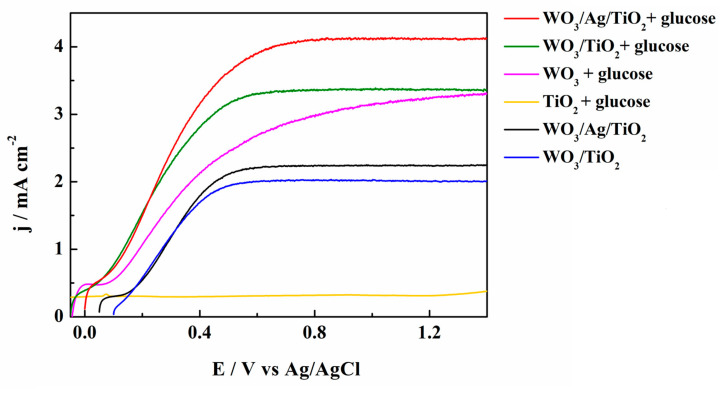
Photocurrent densities vs. imposed potential (j-E) plots in a 0.01 M NaCl/Na_2_SO_4_ electrolyte of pH 7 containing 0.01 M glucose using WO_3_, TiO_2_, WO_3_/TiO_2_ and WO_3_/Ag/TiO_2_ recorded under simulated AM 1.5G irradiation.

**Figure 6:**
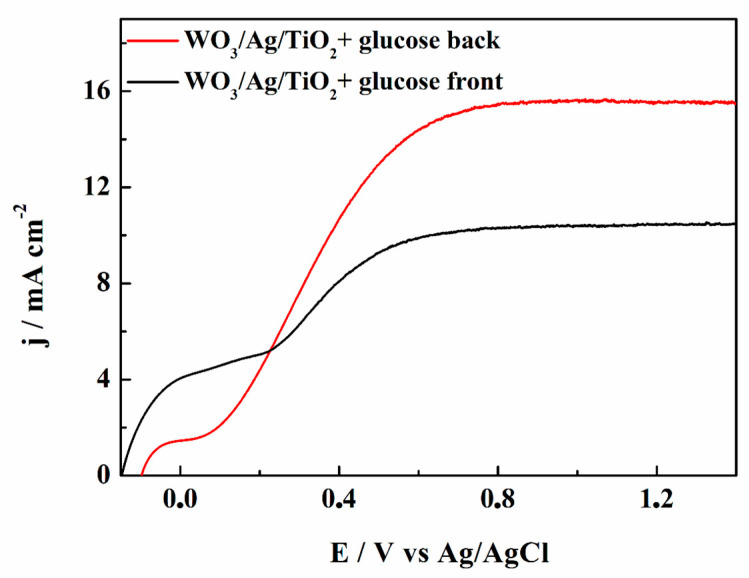
Photocurrent-potential plots for the WO_3_/Ag/TiO_2_ electrode recorded in a 0.01 M NaCl/Na_2_SO_4_ electrolyte of pH 7 with 0.01 M glucose under 3 sun illumination from the front side (black curve) and from the back side (red curve).

In Figure 5, we present photocurrent-potential (j-E) plots for the WO_3_/TiO_2_ and WO_3_/Ag/TiO_2_ photoanodes in a 0.01 M NaCl/Na_2_SO_4_ electrolyte of pH 7. Under simulated solar AM 1.5G (100 mW cm^−2^) irradiation, both photoanodes reached the plateau of ca. 2.2 mA cm^−2^. A table reporting a comparison of our results with the papers cited in the present article is presented in Table S1. The addition of 0.01 mol L^−1^ of glucose to the supporting electrolyte led to very large increases in both photocurrents, with a larger value of ca. 4.1 mA cm^−2^ obtained for the WO_3_/Ag/TiO_2_. This measured value is much higher than the photocurrent obtained for WO_3_/TiO_2_, as well as those for bare WO_3_ and TiO_2_ photoanodes. An interesting result was also provided by measurements performed for the WO_3_/Ag/TiO_2_ photoanode in a 0.01 M NaCl/Na_2_SO_4_ electrolyte of pH 7 with 0.01 mol L^−1^ of glucose under 3 sun illumination (Figure 6), where an impressive increase in the photocurrent, during the backside illumination, up to ca. 16 mA cm^2^ was observed. As mentioned above, this is clearly related to the light reflection by the quasi-continuous layer of silver nanoparticles and by the TiO_2_ overlayer.

**Figure 7:**
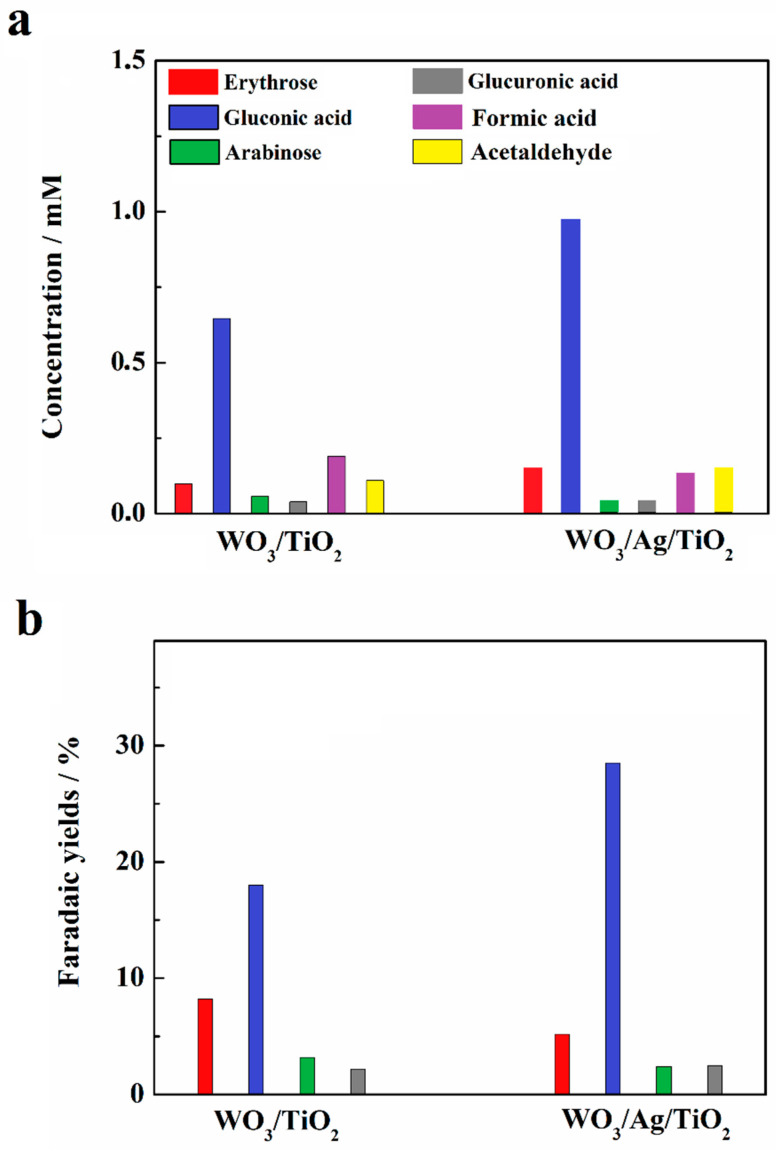
Concentrations (**a**) and Faradaic yields (**b**) of glucose photo-reforming products collected after electrolysis performed following the conditions depicted in the legend of Figure 3.

**Figure 8:**
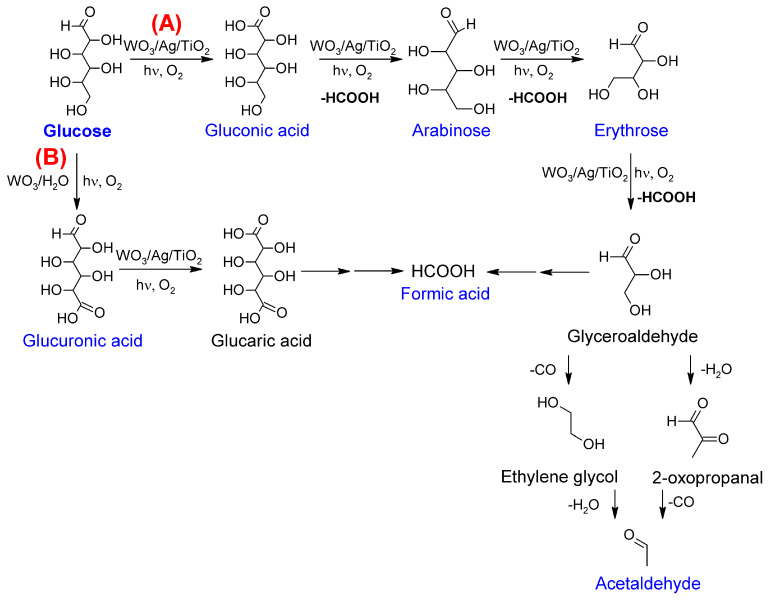
The proposed reaction pathway of glucose reforming over the WO_3_/Ag/TiO_2_ photoanode; products quantified in this work are shown in blue. (**A**) main pathway; (**B**) parallel pathway.

The IPCEs largely exceeding 100% shown in Figure 3 require an additional comment regarding the mechanism of glucose photo-oxidation. As in the case of numerous other organic compounds [25], glucose photo-oxidation involves so-called photocurrent doubling [18]. This means that the initial step of the reaction, involving the transfer of the positive hole generated in the valence band of WO_3_ to the adsorbed molecule of glucose, is followed by the injection, carried out by the formed radical species, of an electron to the conduction band of WO_3_. This corresponds well to the formation of gluconic acid—a two-electron oxidation product. Gluconic acid was by far the most prominent glucose photo-oxidation product obtained after the 5 h long electrolysis, as shown in Figure 4, with much higher Faradaic yields obtained for the WO_3_/Ag/TiO_2_ photoanode (cf. Figure 7b). The presence of significant amounts of arabinose and erythrose in the analyzed post-electrolysis solution suggests that the main glucose photo-reforming pathway was pathway (A) represented in the proposed reaction mechanism shown in Figure 8. We note that the formation of arabinose and erythrose, involving an exchange of two additional electrons, is accompanied by the elimination of formic acid.

As shown in Figure 7, the analyses also revealed other photoelectrolysis products, namely glucuronic acid and acetaldehyde. The formation of acetaldehyde was previously reported during the photo-oxidation of glycerol at TiO_2_-based photocatalysts [26]. The comparison of our analytical data with the literature [7,15,27,28,29,30] led us to identify the pathway (B) as a parallel route for the glucose photo-oxidation reaction (Figure 8).

Analyses of the extent of glucose remaining in the solution at the end of photoelectrolyses showed conversions of 26% and 30%—the larger one was for the WO_3_/Ag/TiO_2_ photoanode. On the other hand, total organic carbon (TOC) analyses indicated the absence of glucose mineralization into carbon dioxide at both photoanodes.

## 4. Conclusions

In summary, we demonstrated the efficient photoelectrochemical oxidation of glucose into principally gluconic acid with a high faradaic yield of almost 30%, achieved using the WO_3_/Ag/TiO_2_ photoanode. The latter photoanode attained much larger photocurrents due to the combination of the optical and electrocatalytic effects induced by the presence of silver. A careful analysis of all formed organic products led us to propose a glucose photo-oxidation mechanism.

## Data Availability

Data are contained within the article and Supplementary Materials.

## Supplementary Materials

The following supporting information can be downloaded at https://www.mdpi.com/article/10.3390/nano14242001/s1, Figure S1. (a) SEM micrographs of Ag nanoparticles on glass/FTO/WO3 substrates deposited with nominal thickness labeled in the left upper corner in red. (b) Histograms of NPs diameter for subsequent nominal thicknesses of Ag layer. Figure S2. The absorbance (Abs) of samples with Ag nanoparticles deposited on glass/FTO/WO3 substrates subtracted from the absorbance (Abs_ref_) of reference samples, i.e., those without nanoparticles. The resonant shape of the presented curves and the nanoparticle size-dependent position of the peaks in the blue spectral region indicate that the observed maxima are associated with LSPR excitation on the silver nanoparticles. Figure S3. Chromatogram of the reacted sample, illustrating the formation of acetaldehyde and formic acid as products of glucose reforming. Figure S4. Chromatogram of the reacted sample, illustrating the formation of monosaccharides and uronic acids as products of glucose reforming. Note that due to co-elution, gluconic acid, and erythrose were quantified using the characteristic fragmentation ions. For the analysis of products, the glucose peak was removed to avoid oversaturating the MS detector. Table S1. PEC performances of the photoanode used in this work compared with those reported by other authors cited in the article. References [31,32] are cited in the supplementary materials.
