# Supplementary material for: Enhanced Visible Light Controlled Glucose Photo-Reforming Using a Composite WO3/Ag/TiO2 Photoanode: Effect of Incorporated Plasmonic Ag Nanoparticles
Source: Nanomaterials (Basel). 2024 Dec 13;14(24):2001. doi: 10.3390/nano14242001 (PMC11728817; doi:10.3390/nano14242001)
Supplement: Supplementary file 1 [file nanomaterials-14-02001-s001.zip › nanomaterials-3296568-supplementary.pdf]

Supplementary Materials

Enhanced visible light controlled glucose photoreforming using a composite $WO_3/Ag/TiO_2$ photoanode: Effect of incorporated plasmonic Ag nanoparticles.

Katarzyna Jakubow-Piotrowska [1,*], Bartlomiej Witkowski [2], Piotr Wrobel [3] Krzysztof Miecznikowski [2] and Jan Augustynski [1,*]

[1] Centre of New Technologies, University of Warsaw, S. Banacha 2c, 02-097 Warsaw, Poland.
[2] Faculty of Chemistry, University of Warsaw, Pasteura 1, 02-093, Warsaw, Poland.
[3] Faculty of Physics, University of Warsaw, Pasteura 5, 02-093 Warsaw, Poland.

* Correspondence: j.augustynski@cent.uw.edu.pl; k.jakubow@cent.uw.edu.pl

S1. Reagents

Sodium tungstate dihydrate, poly(ethylene)glycol (PEG) 300, TiO_2 P25 (containing ca. 75% of anatase and 25% of rutile (Ovonics), poly(vinylidene-fluoride), dimethylformamide, sodium sulfate and sodium chloride were purchased from Sigma-Aldrich. The solutions used in electrochemical measurements were prepared with Mili-Q water. D-(+)-Glucose anhydrous (≥99.0%), D-(-)-arabinose (≥98.0%), D-glucuronic acid (≥97.0%), D-gluconic acid (≥97.0%),D-(+)-galacturonic acid (≥97.0%), D-ribonic acid (≥95.0%), D-(+)-maltose monohydrate (≥99.0%), D-(-)-ribose (≥99.0%), D-(-)-erythrose (≥99.0%), D-xylonic acid (≥95.0%), anhydrous lactose (Pharmaceutical Secondary Standard), trifluoroacetic acid (≥99.0%), ethanetiol (≥97%), isooctane (≥99.5% GCMS grade), sodium hydrogen sulfate monohydrate (≥99.0%), were obtained from Merck (Schnelldorf, Germany). Anhydrous pyridine (reagent grade), N,O-bis(trimethylsilyl)trifluoroacetamide containing 1% of trimethylsilyl chloride (BSTFA+1%TMCS, GC-derivatization grade) were obtained from Chemat Adam Taszner (Grańsk, Poland). Anhydrous ethanol (≥99.5%) was obtained from Avantor Performance Materials (Gliwice, Poland). Ultra-high purity (UHP) gasses: oxygen (used in the total organic carbon analyzer), helium (GC/MS carrier gas), and nitrogen (for sample evaporation) were supplied by Multax (Stare Babice, Poland).

S2. Ag nanoparticles

Localized surface plasmon resonance (LSPR) excited on Ag nanoparticles appears as a characteristic absorbance peak within a material-specific spectral range. Parameters such as width, height, and spectral position of the peak are strongly influenced by the size, shape, and composition of the particles, as well

as the concentration, size distribution of the nanoparticles on the substrate, and the refractive index of the surrounding medium. In general, LSPR redshifts with increasing nanoparticle size and the refractive index of the surrounding medium. Silver spherical nanoparticles with diameters below 20 nm, surrounded by air, exhibit plasmonic resonance in the range of 380–450 nm.

For Ag nanoparticles deposited via the e-beam PVD technique, their size and size distribution are determined by the nominal thickness of the deposited metal layer, while their shape can be adjusted from irregular to quasi-spherical through thermal annealing.

Figure S1a shows SEM micrographs of Ag nanoparticles deposited on a glass/FTO/WO_3 substrate, prepared from silver films with nominal thicknesses of 0.5 nm, 1 nm, 2 nm, and 4 nm, respectively. To achieve a quasi-spherical shape, the nanoisland films were annealed at 150°C for 5 minutes on a standard hot plate. Increasing the nominal thickness of Ag results in nanoparticles with larger average diameters and broader size distributions (Fig. S1b), leading to the following average diameters: 7.39 ± 1.85 nm, 8.77 ± 2.28 nm, 12.42 ± 3.01 nm, and 17.94 ± 4.84 nm for Ag films of 0.5 nm, 1 nm, 2 nm, and 4 nm thickness, respectively.

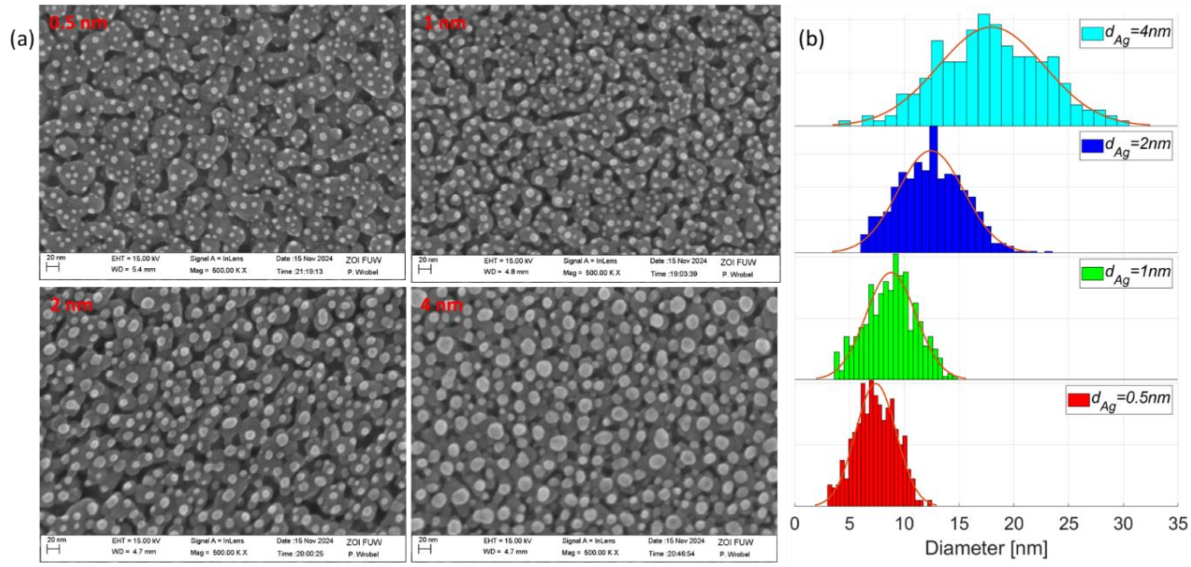

Figure S1. (a) SEM micrographs of Ag nanoparticles on glass/FTO/WO3 substrates deposited with nominal thickness labeled in the left upper corner in red. (b) Histograms of NPs diameter for subsequent nominal thicknesses of Ag layer.

Figure S2 presents the absorbance curves of silver nanoparticle-decorated glass/FTO/WO3 substrates, with the absorbance of bare reference samples subtracted. As expected, the LSPR peak associated with the smallest nanoparticles appears at 386 nm and shifts to longer wavelengths with increasing nanoparticle size, reaching 482 nm for the largest nanoparticles formed from a 4 nm thick Ag film. Additionally, an increase in resonance width is observed, attributed to the broadening of the nanoparticle size distribution as the nominal thickness of the Ag films increases, as shown in Fig. 1b. The overlapping

resonances for 1 nm and 2 nm Ag films may be linked to shape differences, particularly in the latter case, where Ag aggregates tend to form less spherical particles.

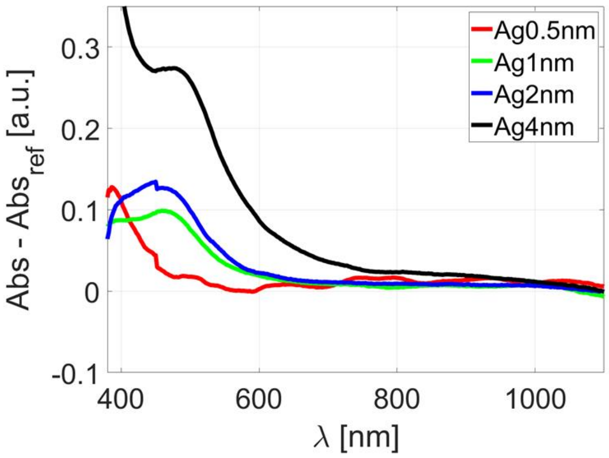

Figure S2. The absorbance (Abs) of samples with Ag nanoparticles deposited on glass/FTO/WO3 substrates subtracted from the absorbance (Abs_{ref}) of reference samples, i.e., those without nanoparticles.

The resonant shape of the presented curves and the nanoparticle size-dependent position of the peaks in the blue spectral region indicate that the observed maxima are associated with LSPR excitation on the silver nanoparticles.

S3. Analysis of total organic carbon

Calibration of this instrument was carried out with the standard solution of glucose in DI water; the concentration range was 5 - 30 mg_{TOC}/L. The linear regression analysis was carried out using the concentration of total organic carbon (TOC) as the independent variable and integrated peak area as the dependent variable; the value of R^2>0.999 was obtained.

Samples from the photoreactor were diluted so that the initial concentration of TOC was approx. 25 mg/L. Samples were then filtered with a syringe filter (hydrophobic PTFE membrane, pore size 0.22 μm) and approx. 3 ml of the solution was placed in the autosampler. Afterward, 50 μl of 2M HCl solution in water was added (by the autosampler) and the sampler was then sparged with pure O_2 to remove the dissolved CO_2 before injection. Uncertainty was calculated as the two values of standard deviation from the triplicate injection of each sample.

S4. Chromatographic analyses

S4.1. Saccharides

For the quantification of saccharides, the autosampler was used in the liquid injection mode, sample volume was 1μl. A capillary column ZB-5MSPlus (Phenomenex): 30m x 0.25mm, 0.25 μm

stationary phase film was used. The column head pressure was 26.7 kPa, the low of the carrier gas was 0.68 ml/min (30 cm/sec), the purge flow was set to 3 ml/min and the split ratio was 10. The analysis conditions were: temperatures of the injector, ion source, and the mass spectrometer transfer line were set to 300°C. The column oven temperature was programmed as follows: the initial temperature was 50 °C for 2 min, then a linear increase at 10 °C/min to 225°C, held for 10 min, then a linear increase at 15 °C/min to 280°C, held for 10 min, the analysis time was 43 min. The MS was operating in the scan mode (45-500 m/z, event time 0.15 s), and analytes were ionized with a beam of electrons (70 eV), the solvent cut was set to 6 min.

S4.2. Acetaldehyde and formic acid

For the quantification of acetaldehyde and formic acid, the autosampler was used in the headspace mode, the sample volume was 0.25 ml. A capillary column 624MS (InterCap): 30m x 0.25mm, 1.4μm stationary phase film, was used. The column head pressure was 47.8 kPa, the flow of the carrier gas was 0.97 ml/min (35.4 cm/sec), the purge flow was set to 3 ml/min and the split ratio was 10. The analysis conditions were: temperatures of the injector: 120 °C, ion source: 140°C, and the mass spectrometer transfer line: 120°C. The column oven temperature was programmed as follows: the initial temperature was 35 °C for 5 min, then a linear increase at 16 °C/min to 150°C, held for 10 min the analysis time was 13 min. The MS was operating in the selected ion monitoring (SIM) mode, m/z values monitored were: 27, 29, 31, 43, 45, 47, 56, 59, 61, 70, 73, 74, and 88, event time 0.15 s. Analytes were ionized with a beam of electrons (70 eV), and the solvent cut was 4.2 min.

S5. Sample preparation

S5.1. Saccharides

For the quantification of glucose, the samples were diluted 20 times, and 3 or 30 μl of this diluted solution (depending on the initial concentration of glucose) was mixed with 35 μl of the internal standard (IS) solution in water (galactose, 1 g/L, which was not detected among the products formed). For the quantification of products, which were all present in much lower concentrations than glucose, 5 or 50 μl of the undiluted sample was added to 35 μl of the same IS solution.

The samples containing the IS were then dried at 60 °C under a gentle stream of N_2. Afterward, saccharides were derivatized accordingly to the previously developed procedure, [1] which involved mercaptalation with ethanethiol/TFA mixture and silylation in pyridine using BSTFA and BSTFA/TMCS (99:1, v/v). The derivatized samples were again dr at 60 °C under a gentle stream of N_2 to remove the leftover solvent (pyridine) and silylating agents, which case GC column degradation, and dissolved in isooctane. 1 μ of the isooctane solution was injected into GC/MS.

S5.2. Acetaldehyde and formic acid

Acetaldehyde and formic acid were derivatized accordingly to the previously described procedure.[2] Brifely 0.5 ml of the undiluted sample was mixed with 2 ml of water, 0.5 ml of ethyl alcohol, and 3.5 g of $NaHSO_4$ in a 10 ml headspace vial, which was then sealed with a PTFE septum. This solution was mixed at 250 RPM at 80°C for 30 min using the GC/MS autosampler and the ethyl esters were injected into the instrument following HS analysis.

Acetaldehyde and formic acid were quantified using a multiple standard addition method, which is often used in the case of samples with complex, concentrated matrices; here all samples contained high amounts of glucose (and other saccharides) and Na_2SO_4 (electrolyte). In the standard addition method, each sample is analyzed multiple times, firstly without adding the standard. Afterward, an increasing amount (mass) of each analyte is added to each sample aliquot and they are analyzed again, under the same experimental conditions. The masses of the individual analytes added to the subsequent samples were between 20 and 50 µg.

S6. Results

S6.1. Quantification of acetaldehyde and formic acid

Sample chromatogram of the reacted sample is shown in Fig. S3.

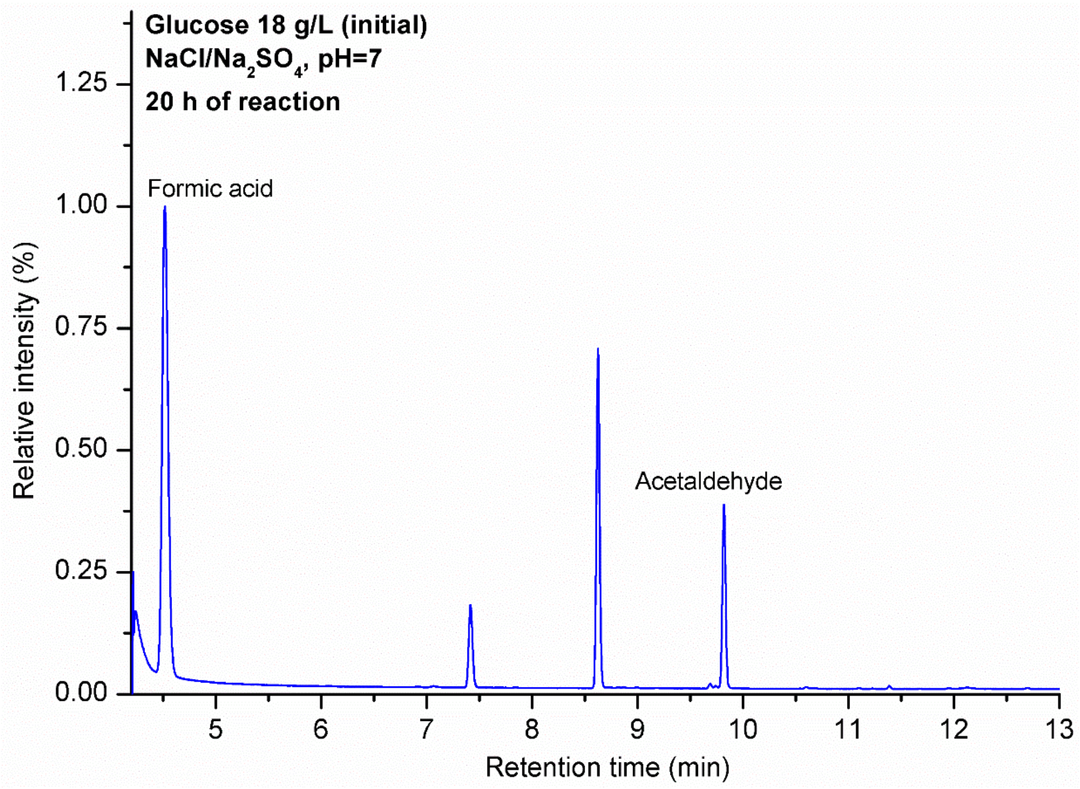

Figure S3 Chromatogram of the reacted sample, illustrating the formation of acetaldehyde and formic acid as products of glucose reforming

S6.2. Quantification of saccharides

Firstly, several commercially-available standards for mono and disaccharides were analyzed to identify the products present in the reacting samples. In all samples, erythrose, gluconic acid, arabinose, and glucuronic acid were identified as products of reforming glucose (Fig S4). For these monosaccharides, calibration was carried out within the range between 0.4 and 6 µg of each analyte (dry mass subjected to derivatization after the evaporation of water). In the case of all compounds under investigation, the linear coefficient of determination (R^2), using the mass of analyte as the interpreted variable and integrated chromatographic peak area as the dependent variable, were all ≥0.999.

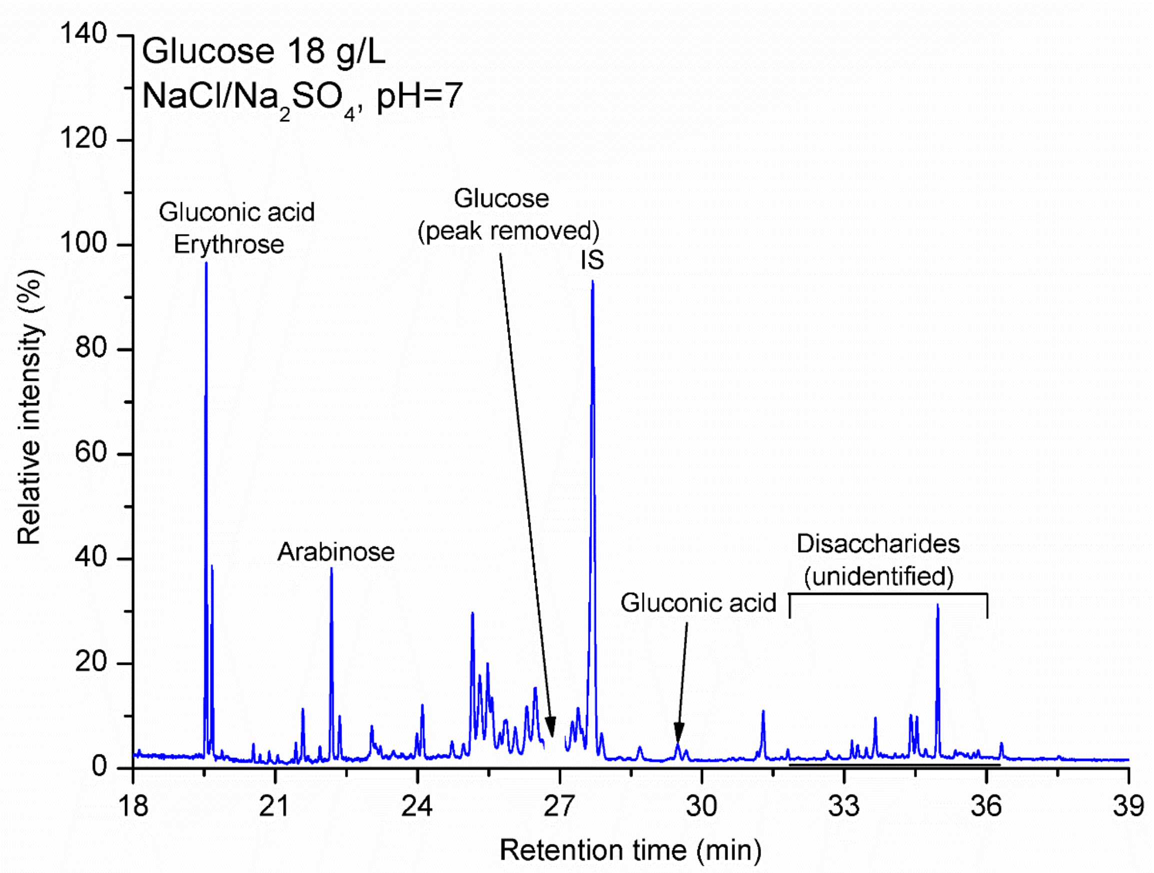

Figure S4 Chromatogram of the reacted sample, illustrating the formation of monosaccharides and uronic acids as products of glucose reforming. Note that due to co-elution, gluconic acid, and erythrose were quantified using the characteristic fragmentation ions. For the analysis of products, the glucose peak was removed to avoid oversaturating the MS detector.

References

31. Colombini, M. P.; Andreotti, A.; Bonaduce, I.; Modugno, F.; Ribechini, E., Analytical strategies for characterizing organic paint media using gas chromatography/mass spectrometry. Acc Chem Res 2010, 43, 715-727.

32. Zhou, D.; Hou, Q.; Liu, W.; Ren, X., Rapid determination of formic and acetic acids in biomass hydrolysate by headspace gas chromatography. Journal of Industrial and Engineering Chemistry 2017, 47, 281-287.

Material	Electrolyte	Photocurrent	Potential	Light source	Detected Products	Ref.
WO_3<br>WO_3/TiO_2	0.5M NaCl pH 4<br>0.5M NaCl pH 7	~ 2.0 mA/cm^2<br>-	at 1.0 V vs Ag/AgCl	AM 1.5G intensity (100 mW cm^{-2})	Gluconic acid<br>Glucuronic acid<br>arabinose<br>erythrose	18
WO_3	0.33M H_2SO_4	~ 0.9 mA/cm^2	at 1.2 V vs SCE	AM 1.5G intensity (100 mW cm^{-2})	CO_2<br>CO	20
single-atom Pt anchored on defective TiO_2 nanorod arrays	1M KOH	~ 1.3 mA/cm^2	at 0.8 V vs RHE	AM 1.5G intensity (100 mW cm^{-2})	Glucaric acid<br>Gluconic acid	21
**$WO_3/Ag/TiO_2$**	**0.01 M NaCl/Na_2SO_4 pH 7**	**~ 2.2 mA/cm^2**	**at 0.6 V vs Ag/AgCl**	**AM 1.5G intensity (100 mW cm^{-2})**	**Gluconic acid<br>Glucuronic acid<br>arabinose<br>erythrose<br>formic acid<br>acealdehyde**	**this work**

Table S1 PEC performances of the photoanode used in this work compared with those reported by other authors cited in the article.
